# Notoginsenoside R1 Protects *db/db* Mice against Diabetic Nephropathy via Upregulation of Nrf2-Mediated HO-1 Expression

**DOI:** 10.3390/molecules24020247

**Published:** 2019-01-10

**Authors:** Bin Zhang, Xuelian Zhang, Chenyang Zhang, Qiang Shen, Guibo Sun, Xiaobo Sun

**Affiliations:** 1 Institute of Medicinal Plant Development, Peking Union Medical College and Chinese Academy of Medical Sciences, Beijing 100193, China; zhangbin7@126.com (B.Z.); zxl2022@126.com (X.Z.); zcy2022@126.com (C.Z.); qshen666@126.com (Q.S.); 2 Key Laboratory of Bioactive Substances and Resources Utilization of Chinese Herbal Medicine, Ministry of Education, Beijing 100193, China; 3 Beijing Key Laboratory of Innovative Drug Discovery of Traditional Chinese Medicine (Natural Medicine) and Translational Medicine, Beijing 100193, China; 4 Key Laboratory of efficacy evaluation of Chinese Medicine against Glycolipid Metabolism Disorder Disease, State Administration of Traditional Chinese Medicine, Beijing 100193, China

**Keywords:** diabetes mellitus, diabetic nephropathy, *db*/*db* mice, HK-2 cells, apoptosis

## Abstract

Diabetic nephropathy (DN) is a leading cause of end-stage renal failure, and no effective treatment is available. Notoginsenoside R1 (NGR1) is a novel saponin that is derived from *Panax notoginseng*, and our previous studies showed the cardioprotective and neuroprotective effects of NGR1. However, its role in protecting against DN remains unexplored. Herein, we established an experimental model in *db*/*db* mice and HK-2 cells exposed to advanced glycation end products (AGEs). The in vivo investigation showed that NGR1 treatment increased serum lipid, β2-microglobulin, serum creatinine, and blood urea nitrogen levels of *db*/*db* mice. NGR1 attenuated histological abnormalities of kidney, as evidenced by reducing the glomerular volume and fibrosis in diabetic kidneys. In vitro, NGR1 treatment was further found to decrease AGE-induced mitochondria injury, limit an increase in reactive oxygen species (ROS), and reduce apoptosis in HK-2 cells. Mechanistically, NGR1 promoted nucleus nuclear factor erythroid 2-related factor 2 (Nrf2) and heme oxygenase-1 (HO-1) expressions to eliminate ROS that induced apoptosis and transforming growth factor beta (TGF-β) signaling. In summary, these observations demonstrate that NGR1 exerts renoprotective effects against DN through the inhibition of apoptosis and renal fibrosis caused by oxidative stress. NGR1 might be a potential therapeutic medicine for the treatment of DN.

## 1. Introduction

Currently, about 425 million individuals are suffering from diabetes mellitus [1]. Microvascular complications induced by diabetes account for a significant number of causalities in diabetes mellitus. Accordingly, diabetic nephropathy (DN) is one of the most devastating diabetic complications and it is the key cause of end-stage renal disease (ESRD) and renal failure [2].

The characteristic pathological alterations of DN involve many aspects, including severe albuminuria, glomerular basement membrane thickening, glomerular and tubular hypertrophy, glomerulosclerosis, and tubulointerstitial fibrosis [2,3]. It is estimated that approximately 15–40% of patients living with type 1 and type 2 diabetes mellitus are affected by DN [4]. In addition to chronic continuous hyperglycemia, other factors including chronic inflammation, dyslipidemia, and accumulation of advanced glycation end products (AGEs) can also markedly contribute to the onset, as well as progression, of DN [5,6].

Chronic hyperglycemia enhances the level of reactive oxygen species (ROS), subsequently facilitating the formation of AGEs [7]. AGEs are implicated in the pathogenesis of DN, which is indicated by the fact that the AGE inhibitors such as aminoguanidine and pyridoxamine ameliorate renal damage in diabetic patients [8]. Numerous researches showed that AGEs not only interact with their receptor and subsequently alter intracellular signaling to promote the release of pro-inflammatory cytokines [9], but also cross-talk with other pathways. Superfluous AGEs in diabetes activate the apoptosis pathway [10], and apoptosis induced by AGEs causes the thickening of the basement membrane and the ultimate loss of glomerular function [11,12]. Therefore, drugs that either suppress AGE formation or attenuate AGEs-induced apoptosis were shown to be renoprotective in an animal model of DN.

Nuclear factor erythroid 2-related factor 2/antioxidant responsive element (Nrf2/ARE) signaling plays key roles in controlling transcriptional regulation of the genes encoding endogenous antioxidant enzymes [13]. Phase II antioxidant enzymes include heme oxygenase-1 (HO-1), nicotinamide adenosine dinucleotide phosphate (NAD(P)H) quinone oxidoreductase-1 (NQO-1), and γ-glutamylcysteine synthetase heavy subunit (γ-GCS). Nrf2-mediated antioxidant enzymes were reported to be promising therapeutic targets for inhibiting oxidative stress and promoting renoprotection [14,15].

**Figure 1:**
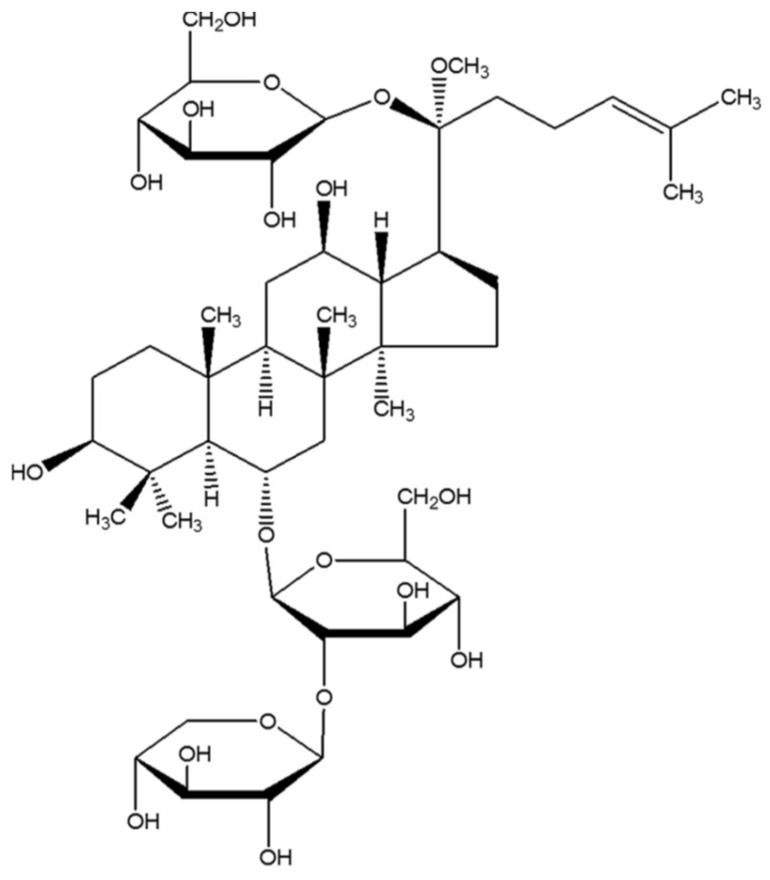
The chemical structure of Notoginsenoside R1 (NGR1).

Notoginsenoside R1 (NGR1, Figure 1) is a novel phytoestrogen isolated from *Panax notoginseng* (*Burk.*) F. H. Chen, an ancient medicinal plant in China that is reported to treat cardiovascular and cerebral vascular diseases [16,17]. Our previous studies showed that NGR1 exerts its neuroprotective role in both H_2_O_2_-induced oxidative damage and amyloid β 25-35-induced neurotoxicity in PC12 cells via blockage of the oxidative stress, apoptosis, and stress-activated mitogen-activated protein kinase (MAPK) signaling pathways [18,19]. Additionally, NGR1 protects against ischemia/reperfusion injuries by regulating oxidative stress- and endoplasmic reticulum stress-related signaling pathways [20]. However, the protective effect of NGR1 on DN is yet to be investigated, and related molecular mechanisms remain unclear.

In this study, transgenic *db*/*db* mice were employed as a type 2 diabetic model, and AGE-treated HK-2 cells were used as an in vitro experimental model to explore the renoprotective mechanisms of NGR1. We find for the first time that NGR1 effectively protects against diabetic nephropathy through activating the Nrf2 pathway and inhibiting apoptosis signaling.

## 2. Results

### 2.1. The Impact of NGR1 on Fasting Blood Glucose (FBG), Total Cholesterol (TCH), and Triacylglycerol (TG) Levels in db/db Mice

**Figure 2:**
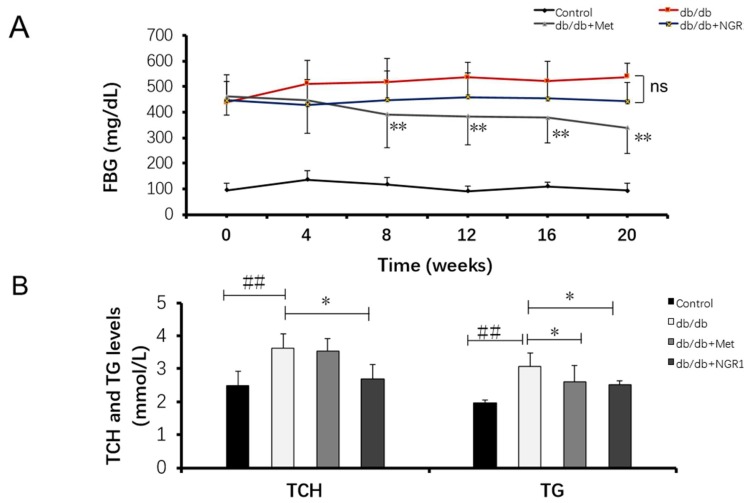
The impact of NGR1 on fasting blood glucose (FBG), total cholesterol (TCH), and triacylglycerol (TG) levels in *db*/*db* mice. (**A**) Fasting blood glucose level in *db*/*db* mice with NGR1 and metformin treatment; (**B**) total cholesterol (TCH) and triglyceride (TG) levels in *db*/*db* mice with NGR1 and metformin treatment. All data are presented as the mean ± SD (*n* = 8). ^##^ *p* < 0.01 vs. the control group; * *p* < 0.05 vs. the model group.

After 20 weeks of treatment with NGR1 and metformin, FBG, TCH, and TG levels were determined. It was found that the impact of NGR1 on FBG was insignificant compared with the model group. Oppositely, metformin could significantly decrease FBG levels in *db*/*db* mice (Figure 2A). NGR1 or metformin could decrease serum TCH and TG levels in *db*/*db* mice compared with the untreated model animals (*p* < 0.05, Figure 2B).

### 2.2. NGR1 Treatment Protected Kidney Function in the db/db Mice

**Figure 3:**
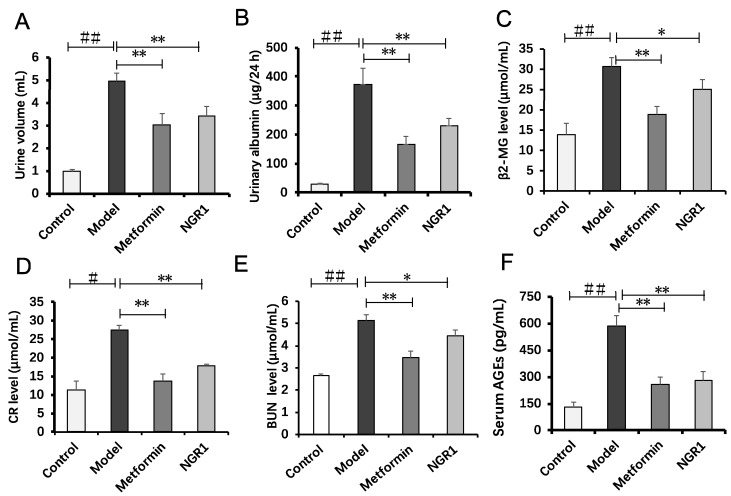
NGR1 ameliorated urinary albumin and biochemical parameters of *db*/*db* mice. (**A**) The 24-h urine volume in various groups; (**B**) urinary albumin level in different groups; (**C**–**F**) serum levels of β2-microglobulin (β2-MG), creatinine (CR), blood urea nitrogen (BUN), and advanced glycation end products (AGEs) in *db*/*db* mice. All data are presented as the mean ± SD (*n* = 8). ^##^ *p* < 0.01 vs. the control group; * *p* < 0.05 or ** *p* < 0.01 vs. the model group.

Compared with the control mice, 24-h urine volume and urinary albumin levels in diabetic *db*/*db* mice were significantly high (Figure 3A, total urine volume: *db*/*db*: 4.95 ± 0.35 vs. db/m: 0.99 ± 0.08; Figure 3B, urinary albumin: *db*/*db*: 374.16 ± 55.32 vs. 29.20 ± 1.89, *p* < 0.01). Metformin and NGR1 treatment for 20 weeks markedly reduced 24-h urine volume and albumin levels (Figure 3A,B, *p* < 0.01).

As β2-microglobulin (β2-MG), creatinine (CR), and blood urea nitrogen (BUN) levels are also considered as markers of renal function in the clinic, they were determined in this study to evaluate renal function. As shown in Figure 3C–E, levels of β2-MG, CR, and BUN in the model group were significantly increased by 115.31% (*p* < 0.01), 143.48% (*p* < 0.01), and 93.11% (*p* < 0.01), respectively, in comparison with the control group, indicating that renal function in diabetic *db*/*db* mice was remarkably impaired. The inhibitive effect of metformin on the increased β2-MG, CR, and BUN levels was slightly better than that of NGR1. With 20 weeks of NGR1 treatment, β2-MG, CR, and BUN levels were lowered by 22.93% (*p* < 0.05), 54.07% (*p* < 0.05), and 15.33% (*p* < 0.05), respectively. Additionally, both metformin and NGR1 treatment significantly inhibited the serum AGE increase in *db*/*db* mice (Figure 3F). These results collectively indicated that NGR1 and metformin possessed the capacity to prevent renal dysfunction in diabetic mice.

### 2.3. The Impact of NGR1 on Histopathological Changes

**Figure 4:**
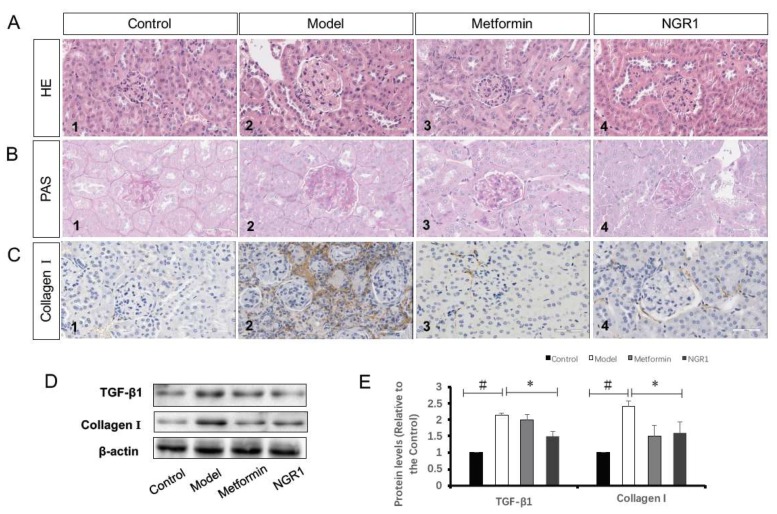
NGR1 attenuated histopathological changes via inhibition of transforming growth factor beta (TGF-β1)-mediated collagen I expression. Representative images of hematoxylin and eosin (H&E) staining (**A**) and periodic acid–Schiff base (PAS) staining (**B**) for pathological structure of kidneys in *db*/*db* mice (magnification: 400×); (**C**) representative images of collagen I determined by immunohistochemistry (ICH) staining (magnification: 400×); (**D**) representative images of TGF-β1 and collagen I protein expression determined by Western blot in the kidneys of each group; (**E**) relative protein expressions of TGF-β1 to β-actin, and collagen I to β-actin are expressed in the bar graphs. All data are presented as the mean ± SD (*n* = 3). ^#^ *p* < 0.05 vs. the control group; * *p* < 0.05 vs. the model group.

Hematoxylin and eosin (H&E) stains showed that the structure of the glomerulus in the diabetic model group exhibited marked abnormalities, including an increase in glomerular volume and significantly increased mesangial cell proliferation compared to those in the control group. Additionally, a substantial inflammatory infiltration area could be found in kidney sections from the diabetic *db*/*db* mice (Figure 4A1). Oral administration of NGR1 and metformin alleviated structural abnormalities in the kidney of *db*/*db* mice (Figure 4A3,4).

Periodic acid–Schiff base (PAS) staining showed that the basement membrane of glomerular capillaries in the kidney from the *db*/*db* mice thickened, the glycogen and mesangial matrix increased in the mesangial region, and the mesangial area widened (Figure 4B2). In the kidney slides from metformin- and NGR1-treated *db*/*db* mice, less glycogen was deposited in the mesangial region (Figure 4B3,4). Results from immunohistochemistry (ICH) also showed that both metformin and NGR1 decreased collagen I expression in diabetic kidney tissue (Figure 4C). Furthermore, Western blot analysis was employed to determine protein levels of collagen I, as well as of transforming growth factor beta (TGF-β1). As shown in Figure 4C, diabetes promoted the expression of both TGF-β1 and collagen I compared to the control group (2.15- and 2.42-fold of control, respectively, *p* < 0.05). NGR1 treatment significantly downregulated the increased TGF-β1 and collagen I protein levels (*p* < 0.05), indicating that NGR1 suppressed pathological changes of DN via inhibition of the TGF-β pathway.

### 2.4. NGR1 Inhibits Diabetes-Induced Apoptosis via Upregulation of Nrf2-Mediated HO-1 Expression

**Figure 5:**
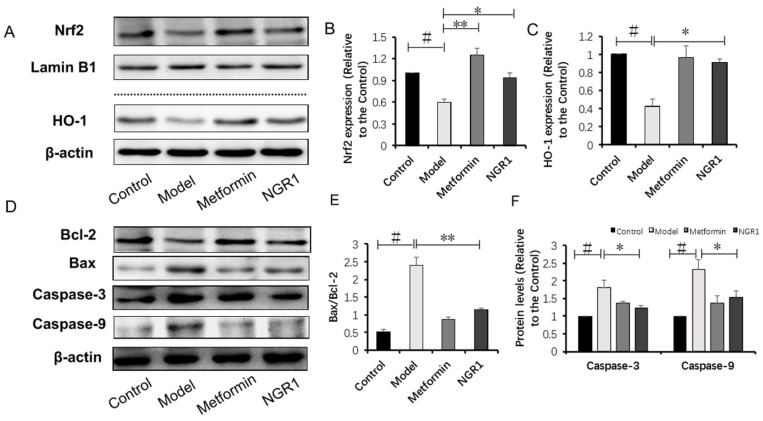
NGR1 inhibited apoptosis via upregulation of nuclear factor erythroid 2-related factor 2 (Nrf2)-mediated heme oxygenase-1 (HO-1) expression in *db/db* mice. (**A**) Representative images of nuclear Nrf2 and HO-1 protein expression determined by Western blot; (**B**,**C**) relative protein expressions of nuclear Nrf2 to Lamin B1, and HO-1 to β-actin are expressed in the bar graphs; (**D**) representative images of apoptotic protein immunoblots and (**E**,**F**) immunoblotting results quantified in the bar graphs. All data are presented as the mean ± SD (*n* = 3). ^#^ *p* < 0.05 vs. the control group; * *p* < 0.05 or ** *p* < 0.01 vs. the model group.

Cell apoptosis is closely associated with the activated TGF-β pathway, and it plays key roles in the development of DN [21,22]. A terminal deoxynucleotidyl transferase (TdT) deoxyuridine triphosphate (dUTP) nick-end labeling (TUNEL) assay was performed to detect apoptotic cells, and showed that apoptotic cells in the model group markedly increased compared with those in the control, which were significantly reduced by metformin and NGR1 treatment (Figure S1). Western blot analysis also showed that the dramatic increase in B-cell lymphoma 2 (Bcl-2)-associated X protein (Bax)/Bcl-2 ratio, caspase-3, and caspase-9 expression in diabetic mice was reduced by NGR1 treatment for 20 weeks (Figure 5D–F).

Nrf2-mediated antioxidant enzymes possess the capacity to repress apoptosis [23]. NGR1, as a novel phytoestrogen, is reported to promote translocation of Nrf2 to subsequently upregulate HO-1. In accordance with previous studies [24], nucleus Nrf2 and HO-1 expressions were markedly decreased in diabetic mice compared to those of the control group (Figure 5A,B). The decreased nucleus Nrf2 and HO-1 levels in diabetic mice could be conspicuously reversed by administration with metformin (Figure 5A,B) and NGR1 (Figure 5A,C) when compared with the model group (*p* < 0.05), indicating that Nrf2 mediated antioxidant signaling to exert its anti-apoptotic roles.

### 2.5. NGR1 Inhibits AGE-Induced HK-2 Cell Death

**Figure 6:**
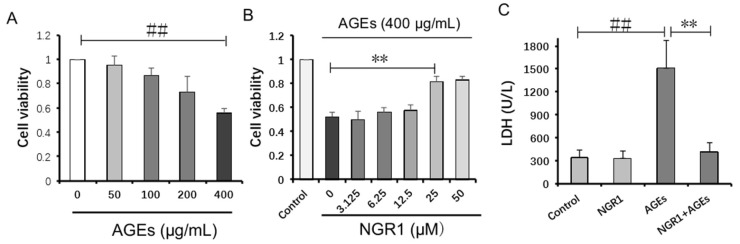
NGR1 attenuated AGE-induced HK-2 cell death. (**A**) HK-2 cell viability was reduced by incubation with AGEs for 24 h; (**B**) NGR1 attenuated HK-2 cell death induced by AGEs; (**C**) NGR1 decreased HK-2 cell lactate dehydrogenase (LDH) release induced by AGEs. All data are presented as the mean ± SD of three independent experiments (*n* = 9). ^##^ *p* < 0.01 vs. the control group; ** *p* < 0.01 vs. the AGE group.

To study the protective effects of NGR1 on AGE-induced HK-2 cell death, we firstly evaluated the general toxicity of AGEs. The HK-2 cells were incubated with a series of AGE concentrations (0, 50, 100, 200, and 400 μg/mL) for 24 h. Cell viability was measured using the 3-(4,5-dimethylthiazol-2-yl)-2,5-diphenyltetrazolium bromide (MTT) assay. As shown in Figure 6A, cell viability was reduced to 55.74 ± 4.08% when treated with 400 μg/mL AGEs for 24 h. Therefore, 400 μg/mL AGEs were used in the subsequent experiments. Subsequently, the potential protective effects of NGR1 against AGE-induced HK-2 cell injury were assessed. As shown in Figure 6B, cell viability increased to 81.06 ± 4.64% with pretreatment with 25 μmol/L NGR1 compared with exposure to only 400 μg/mL AGEs for 24 h. Accordingly, the optimal concentration and pretreatment time with NGR1 was determined to be 25 μmol/L and 24 h, respectively. Lactate dehydrogenase (LDH) leakage, used as a biomarker of cell death, was also detected. AGE treatment significantly increased the LDH leakage compared with the control group, and NGR1 preconditioning effectively decreased the LDH release (Figure 6B, *p* < 0.01). These results collectively indicated that NGR1 could protect HK-2 cells against AGE-induced cell death.

### 2.6. NGR1 Suppresses AGE-Induced Mitochondrial Superoxide Production in HK-2 Cells via Upregulation of Nrf2-Mediated HO-1

**Figure 7:**
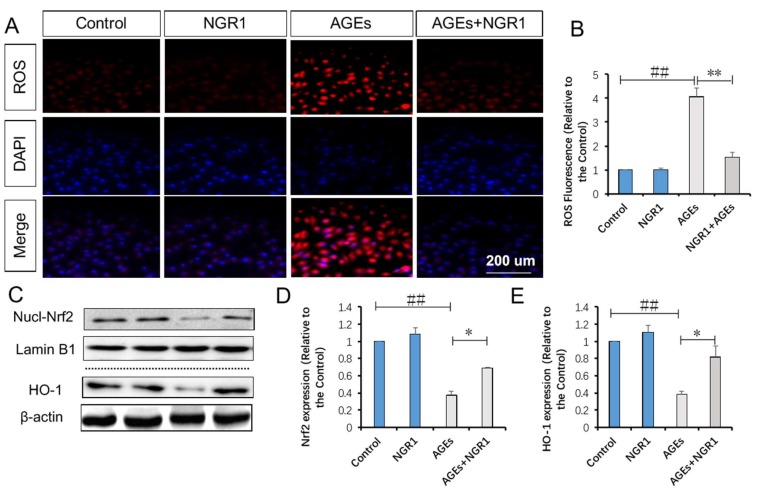
NGR1 inhibited intracellular reactive oxygen species (ROS) production in HK-2 cells exposed to AGEs via upregulation of Nrf2-mediated HO-1 expression. (**A**) Fluorescence images and (**B**) the bar diagram show ROS levels in HK-2 cells; the fluorescence intensity of ROS was measured using a fluorescence microplate reader, where the bar represents 200 μm; (**C**) representative images of nuclear Nrf2 and HO-1 protein expression determined by Western blot; (**D**–**E**) relative protein expressions of nuclear Nrf2 to Lamin B1, and HO-1 to β-actin are expressed in the bar graphs. All data are presented as the mean ± SD (*n* = 3). ^##^ *p* < 0.01 vs. the control group; * *p* < 0.05 or ** *p* < 0.01 vs. the AGE group.

A previous study showed that AGEs increase reactive oxygen species (ROS) production and result in cell apoptosis [25]. Thus, we detected ROS production in response to AGE simulation and NGR1 intervention. Intracellular ROS exhibits red fluorescence under the microscope. In normal HK-2 cells, few showed red fluorescence, indicating that intracellular ROS levels were low. The exposure to AGEs led to a significant increase in intracellular ROS production (4.07-fold of the control group, *p* < 0.01), which could be markedly alleviated by pretreatment with 25 μmol/L NGR1 (1.55-fold of the control group, *p* < 0.01, Figure 7A,B).

Nrf2 signaling is implicated in fighting against oxidative stress and we examined the impact of NGR1 on Nrf2-regulated antioxidant enzyme, HO-1. Western blot analysis showed that AGEs significantly inhibited nucleus Nrf2 expression, and that the inhibitive effect of Nrf2 could be reversed by pretreatment of HK-2 cells with NGR1 (Figure 7C,D). Consistent with Nrf2 changes, NGR1 remarkably attenuated the suppression of HO-1 expression induced by AGEs (*p* < 0.05, Figure 7C,E). These results collectively demonstrated that NGR1 plays a potent protective role in the suppression of oxidative stress via the regulation of Nrf2 signaling.

### 2.7. NGR1 Alleviates AGE-Induced Mitochondrial Injury in HK-2 Cells

**Figure 8:**
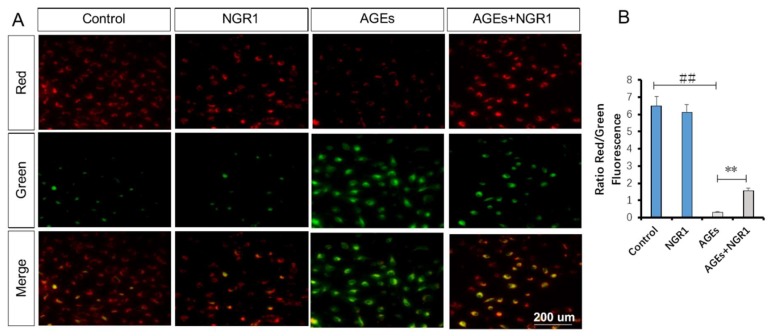
NGR1 inhibited AGE-induced mitochondrial transmembrane potential (MTP) reduction in HK-2 cells. (**A**,**B**) Representative images and quantitative analysis of JC-1 staining. Treating HK-2 cells with AGEs caused a significant decrease in the ratio of red to green fluorescence intensity, which is a sign of the early stages of cell apoptosis. The bar represents 200 μm. All data are presented as the mean ± SD (*n* = 10 wells per group). ^##^ *p* < 0.01 vs. the control group; * *p* < 0.05 vs. the AGE group.

JC-1 staining was used to detect the disruption of mitochondrial transmembrane potential (MTP), one of the early events in the mitochondrial pathway activation of apoptosis. Mitochondria in normal HK-2 cells emits red fluorescence with JC-1 staining. AGEs caused an increase in green fluorescence in HK-2 cells, indicating the depolarization of MTP. NGR1 preconditioning restored the decreased MTP compared with the MTP in cells exposed to AGEs (1.54 vs. 0.32, *p* < 0.01, Figure 8B).

### 2.8. NGR1 Inhibits AGE-Induced Cell Apoptosis in HK-2 Cells

**Figure 9:**
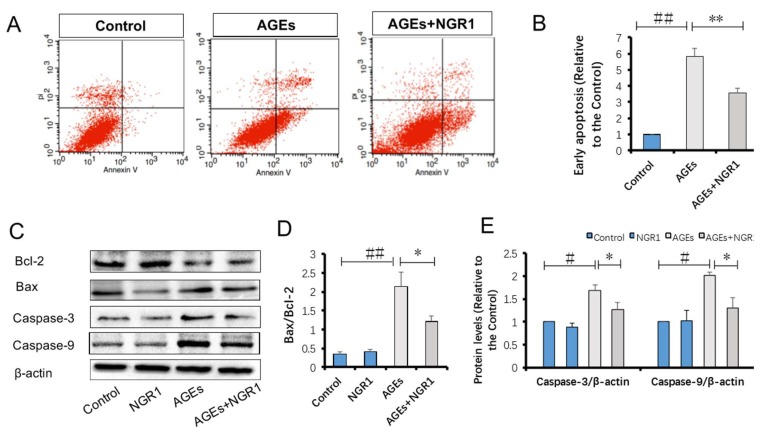
NGR1 suppressed HK-2 cell apoptosis induced by AGEs; (**A**,**B**) annexin V/propidium iodide (PI) staining, quantitated by flow cytometry analysis; (**C**) Western blot analysis of B-cell lymphoma 2 (Bcl-2), Bcl-2-associated X protein (Bax), caspase-3, and caspase-9 expressions; (**D**) relative expression levels of Bax/Bcl-2, caspase-3, and caspase-9 compared to β-actin are expressed in the bar graphs. All data are presented as the mean ± SD (*n* = 3). ^#^ *p* < 0.05 or ^##^ *p* < 0.01 vs. the control group; * *p* < 0.05 or ** *p* < 0.01 vs. the AGE group.

Annexin V/propidium iodide (PI) staining was performed to detect the protective role of NGR1 in AGE-induced HK-2 cell apoptosis. Quantitative analysis using flow cytometry showed that AGE treatment significantly increased apoptosis in HK-2 cells by almost 5.84-fold compared with the control group; NGR1 preconditioning markedly reduced this increase to a 3.54-fold change (*p* < 0.01, Figure 9A,B).

Western blot analysis was further performed to assess proteins involved in the mitochondrial pathway of apoptosis. NGR1 preconditioning reversed the effects of AGEs by increasing Bcl-2 and decreasing Bax expression levels. Similarly, both caspase-3 and caspase-9 were also upregulated in HK-2 cells treated by AGEs, and their expression levels were downregulated with NGR1 pretreatment (*p* < 0.05, Figure 9C–E). Thus, these results indicated that NGR1 could upregulate anti-apoptotic proteins and downregulate pro-apoptotic proteins.

### 2.9. HO-1 Inhibitor Neutralizes the Protective Effects of NGR1 by Resisting Its Inhibition of Apoptosis and the TGF-β Pathway

**Figure 10:**
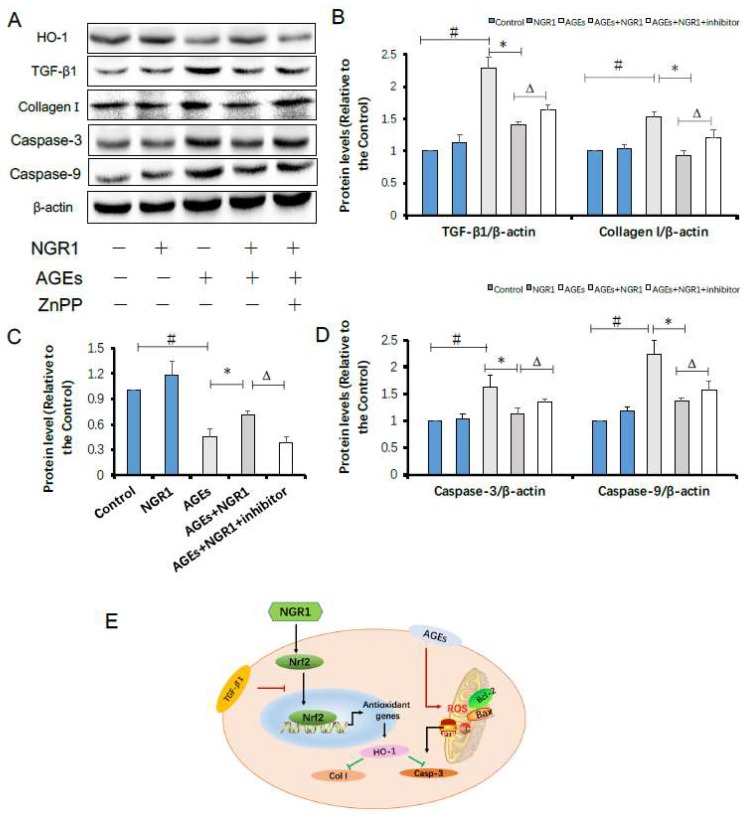
The protective effects of NGR1 on HK-2 cells exposed to AGEs were neutralized by HO-1 inhibitor (zinc protoporphyrin (ZnPP)); (**A**) representative immunoblots of the protein expressions of HO-1, TGF-β1, collagen I, caspase-3, and caspase-9; (**B**–**D**) relative expression levels of HO-1, TGF-β1, collagen I, caspase-3, and caspase-9 compared to β-actin are expressed in the bar graphs. All data are presented as the mean ± SD (*n* = 3). ^#^ *p* < 0.05 vs. the control group; * *p* < 0.05 vs. the AGE group; ^∆^ *p* < 0.05 vs. the AGE + NGR1 group; (**E**) schematic illustration showing the mechanisms underlying the prevention of diabetes/AGE-induced damage in HK-2 cells and *db*/*db* mice.

The results above hinted to us that HO-1 regulated by Nrf2 might be involved in the protection of NGR1 against diabetes-induced apoptotic and fibrotic pathway. Therefore, zinc protoporphyrin (ZnPP), an HO-1 inhibitor, was used to explore this hypothesis. As expected, after HO-1 inhibition, both the expressions of fibrotic proteins, including TGF-β1 and collagen I, and the expressions of apoptosis proteins, including caspase-3 and caspase-9, increased again (Figure 10A–D), indicating that the anti-fibrotic and anti-apoptotic effects of NGR1 on HK-2 cells under AGE conditions were neutralized (Figure 10E). These results collectively showed that NGR1 activates Nrf2-mediated antioxidant signaling to restrict diabetic apoptosis and fibrosis.

### 2.10. Discussion

Diabetic nephropathy (DN) is a progressive microvascular complication occurring among diabetic patients, characterized by renal microvasculature disruption, and glomerular capillary and tubular interstitium damage [26]. Accumulating studies indicated that oxidative stress induced by chronic hyperglycemia can trigger the degenerative pathways leading to DN [26,27]. Oxidative stress not only leads to podocyte apoptosis, an important factor in the pathogenesis of proteinuria in DN [28], but also promotes glomerular mesangial cell (MC) proliferation and excessive extracellular matrix (ECM) protein production [29,30]. In the present study, we demonstrated that NGR1 showed protective effects on DN both in diabetic *db*/*db* mice and in HK-2 cells treated with AGEs. The *db*/*db* mice exhibited typical disordered features including increased levels of FBG, TG, TCH, BUN, and CR, as well as renal structural abnormalities. The results presented here demonstrated that NGR1 could decrease the levels of urinary albumin, and serum TG, TCH, BUN, CR, and β2-MG levels in *db*/*db* mice, alleviate the pathological changes of the kidney, and downregulate the expression levels of TGF-β1 and collagen I, clearly exhibiting a certain protective effect on diabetic nephropathy. Additionally, NGR1 inhibited oxidative stress and apoptosis through promoting Nrf2-mediated HO-1 expression both in diabetic mice and in HK-2 cells exposed to AGEs.

It is well known in the clinic that increased proteinuria is a key hallmark of glomerular injury and an important manifestation of progressive kidney damage in patients with DN [31]. In line with previous reports [32], diabetic *db*/*db* mice in the model group showed elevated urinary albumin, β2-MG, CR, and BUN levels, which were mainly induced by the pathological changes in the kidneys, including glomerular capillary wall thickening, extracellular matrix deposition, and the expansion and proliferation of mesangial cells [33]. In this study, we found NGR1 treatment significantly inhibited an increase in glomerular volume and mesangial cell proliferation in diabetic *db*/*db* mice (Figure 4A). Structurally, mesangial matrix expansion is caused by superfluous deposition of extracellular matrix (ECM) proteins in the mesangial area [34]. Excessive synthesis of ECM proteins subsequently leads to glomerular sclerosis with renal dysfunction. PAS stains showed that conspicuous fibrosis was found in diabetic kidneys compared to the control group. In contrast, the fibrotic alterations in the kidney were dramatically improved by NGR1 treatment, indicating that administration of NGR1 may alleviate the structural abnormalities in the kidneys of *db*/*db* mice.

Numerous studies showed that TGF-β signaling is a key pathway underlying DN development and it is activated in the glomeruli of diabetic patients and animal models [35]. Hiroyuki found that AGEs could induce activation of TGF-β signaling [36]. Results from Western blot showed that TGF-β1 and collagen I expressions increased in both diabetic kidneys and in HK-2 cells exposed to AGEs, the expressions of which were downregulated by NGR1 treatment, indicating that NGR1 suppresses diabetes-induced fibrosis via inhibition of the TGF-β pathway.

In the past decades, great progress was achieved in the treatment of diabetes complications. Diabetic nephropathy is reported to be correlated with excess AGEs due to abnormal metabolic function in diabetes. Overproduction of AGEs both in diabetes patients and in animal models is a risk factor for DN [37,38], which leads to severe oxidative stress. As AGE-induced structural and functional injury in kidney is independent of hyperglycemia [39], only blood-glucose control could barely inhibit the progress of DN [40]. The accumulation of AGEs plays a crucial role in the onset and development of DN. Accordingly, AGEs were employed to induce HK-2 cell injury. It was found that 400 μg/mL AGEs significantly reduced HK-2 cell viability and increased LDH leakage from cells; these observations were reversed by NGR1 treatment (Figure 6C), indicating that NGR1 plays a protective role through attenuating AGE-induced injury.

Growing evidence indicates that oxidative stress is a key pathogenesis contributing to DN [41]. Oxidative stress can cause deterioration of the glomerular endothelial surface layer, leading to exacerbation of glomerular permselectivity and development of albuminuria [42]. Previous findings showed that AGEs significantly increase ROS levels via activation of NADPH oxidase, which plays an important part in the generation of ROS [43]. In accordance with previous studies, AGEs markedly increased ROS production, which could be eliminated by pretreatment with NGR1, hinting to us that NGR1 could attenuate oxidative stress induced by AGEs.

Oxidative stress is closely associated with apoptosis in the kidneys of diabetic patients or mice [44]. Given that NGR1 ameliorated AGE-induced oxidative stress, we hypothesized that it subsequently suppressed the apoptosis of HK-2 cells. Structural and functional abnormality of mitochondria is directly related to apoptosis. In this study, AGEs caused loss of mitochondrial membrane potential (MTP), which was restored by NGR1 treatment. Annexin V/PI staining also indicated that NGR1 could inhibit early apoptosis induced by AGEs. The apoptosis pathways were further studied to explore the molecular basis of the effects of NGR1. Both in vivo (Figure 5D,F) and in vitro (Figure 9C,E) experiments showed that expressions of apoptotic proteins, including caspase-3 and caspase-9, markedly increased in diabetic kidneys or HK-2 cells treated with AGEs. Bcl-2 family proteins are reported to be vital mediators of apoptotic processes [45]. Bcl-2 and Bax control the mitochondria permeability [46]. Additionally, overproduction of ROS increases Bax/Bcl-2 ratio, which can decease MTP and subsequently mediate caspase-3 expression [47,48]. Consistent with these data, NGR1 treatment reduced the expression of pro-apoptotic Bax but promoted the expression of anti-apoptotic Bcl-2. These results collectively indicated that NGR1 plays its anti-apoptotic roles by eliminating ROS production and restoring MTP.

Estrogen receptor was reported to promote Nrf2 signaling via the phosphoinositide 3-kinase (PI3K)/protein kinase B (AKT) pathway [49]. Previously, we found that NGR1 could regulate the estrogen receptor-mediated pathway, protecting against diabetic cardiomyopathy [50]. Thus, we speculated that this agent might have an impact on heme oxygenase-1, an endogenous cytoprotective enzyme with antioxidative and anti-apoptotic properties, which plays a key role in attenuating oxidative stress [51]. In addition, studies showed that activation of nuclear Nrf2 can promote HO-1 expression [52]. It was clarified that AGE-induced ROS block the translocation of Nrf2 into the nucleus, inhibiting HO-1 expression [53]. In the development of DN, AGEs induce the expressions of fibronectin and TGF-β1, which are attenuated by activation of the Nrf2 pathway [54], indicating that cross-talk exists between TGF-β and the Nrf2 pathway. Therefore, how to activate Nrf2-mediated signaling and antioxidant enzymes remains meaningful for the treatment of DN. In the present study, we confirmed that nuclear Nrf2 and HO-1 expressions were significantly downregulated both in diabetic kidneys (Figure 5A–C) and in HK-2 cells treated with AGEs (Figure 7C–E), which could be reversed by NGR1 intervention. To confirm whether NGR1 exerted its protective roles via Nrf2-mediated HO-1, an HO-1 inhibitor (ZnPP) was used in this study. In expectation, the aforementioned anti-apoptotic effects of NGR1 were abolished by the addition of ZnPP, suggesting that the antioxdidant and anti-apoptotic effects of this agent were related to the Nrf2 pathway and HO-1 expression (Figure 10E).

## 3. Materials and Methods

### 3.1. Reagent and Materials

Notoginsenoside R1 (NGR1, molecular weight = 933.14, purity >98.6) was obtained from Shanghai Winherb Medical S&T Development (Shanghai, China). Metformin (Sino-American Shanghai Squibb Pharmaceuticals Ltd. Shanghai, China) was used as the positive control in this study. All cell culture materials, Dulbecco’s modified Eagle’s medium (DMEM), fetal bovine serum (FBS), and penicillin/streptomycin, were obtained from Gibco (Shanghai, China). Furthermore, 3-[4,5-dimethylthylthiazol-2-yl]-2,5 diphenyltetrazolium bromide (MTT), zinc protoporphyrin (ZnPP), and other chemicals were purchased from Sigma-Aldrich (St. Louis, MO, USA). The AGE determination kit was purchased from Shanghai Westang Bio-tech co., Ltd. Primary antibodies against collagen I (ab34710), HO-1 (ab13248), caspase-3 (ab4051), and caspase-9 (ab32539) were obtained from Abcam (Cambridge, UK). Primary antibodies against TGF-β1 (sc-146), Nrf2 (sc-13032), Lamin B1 (sc-6217), Bcl-2 (sc-7382), and Bax (sc-4239) were purchased from Santa Cruz Biotechnology (Santa Cruz, CA, USA). Horseradish peroxidase (HRP)-labeled goat anti-rabbit immunoglobulin G (IgG) (H + L), HRP-labeled goat anti-mouse IgG (H + L), and β-actin antibody were obtained from Beyotime Institute of Biotechnology (Shanghai, China).

### 3.2. Preparation of AGE/Bovine Serum Albumin (BSA)

AGE-BSA was prepared according to the protocol of Zhang et al. [55]. Briefly, 0.07 g of bovine serum albumin in phosphate-buffered saline (PBS) was incubated with 0.7926 g of d-glucose at 37 °C for eight weeks. Control albumin was incubated without glucose. Endotoxin was removed using a Pierce endotoxin removing gel and was determined using a ToxinSensor TM chromogenic LAL Endotoxin Assay Kit (GenScript, Piscataway, NJ, USA), which was less than 500 U/L.

### 3.3. Animal and Groups

Both female C57BL/KsJ *db*/*db* mice (*db*/*db* mice) and C57BL/6J mice (db/m, 6–8 weeks old) were purchased from from the Model Animal Research Center of Nanjing University (Nanjing, China). The mice were maintained under standard laboratory conditions (room temperature at 22 °C, humidity of 60% with a 12 h light/dark cycle) and fed with a standard pellet diet and water ad libitum. All animal experiments were approved by the Experimental Laboratory Animal Committee of Chinese Academy of Medical Sciences and Peking Union Medical College and were performed in accordance with the guidelines of the National Institutes of Health Guide for the Care and Use of Laboratory Animals published by the United States (US) National Institutes of Health (NIH Publication No. 85–23, revised 1996). All sacrifices were performed under pentobarbitone anesthesia, and every effort was made to minimize animal suffering.

### 3.4. Experimental Protocol

After two weeks of adaptation, tail blood glucose levels were measured using a glucometer (Roche). The *db*/*db* mice with fasting blood glucose >200 mg/dL were considered diabetic and were used for the further experimentation. The mice were randomly divided into four groups (*n* = 8): (1) C57BL/6 group (control); (2) *db*/*db* group (model); (3) *db*/*db* + metformin 200 mg/kg/day group (Met); (4) *db*/*db* + NGR1 30 mg/kg/day group. NGR1 or metformin was fed to the mice via gavage every day for 20 weeks. Mice in the control and model groups were gavaged with a vehicle. After 20 weeks of treatment, urine volume was measured by placing animals in metabolic cages.

### 3.5. Fasting Blood Glucose, and Serum TCH and TG Levels

Fasting blood glucose (FBG) levels of overnight fasted mice were measured every four weeks. After 20 weeks of treatment, the animals were sacrificed by cervical dislocation. Before sacrificing, the blood samples were collected from retro-orbital venous plexus for serum total cholesterol (TCH) and triacylglycerol (TG) determination using biochemical kits (BioSino Bio-Technology & Science Inc. Beijing, China).

### 3.6. Serum β2-Microglobulin, Blood Urea Nitrogen (BUN), Creatinine (CR), AGE, and Urinary Albumin Measurements

Serum samples were separated from blood samples by centrifuging (1000× *g*, 10 min, 4 °C). β2-MG, BUN, CR, and AGE levels were assayed using a multi-mode microplate reader (Synergy HI, BioTek, Winooski, VT, USA) and commercially available kits (Jiancheng Bioengineering Institution, Nanjing, China) according to the manufacturers’ instructions. Urinary albumin was measured using an Albumin (Mouse) Elisa Kit according to the manufacturer’s instructions (KA0489, Abnova, Taibei, Taiwan). The urinary albumin excretion was expressed by the total amount excreted in 24 h.

### 3.7. Histopathological Examination

The kidney samples were fixed in 4% buffered paraformaldehyde, dehydrated, and then embedded in paraffin. Sections of about 4 μm thickness were sliced from each embedded sample and stained with hematoxylin and eosin (H&E) or periodic acid–Schiff base (PAS), before being examined using a light microscope by a pathologist who was blinded to the groups under study.

### 3.8. Terminal Deoxynucleotidyl Transferase (TdT) dUTP Nick-End Labeling (TUNEL) Staining

DNA fragmentation in kidney apoptotic cells was determined by TUNEL staining according to the manufacturer’s instructions (Roche, Mannheim, Germany). Briefly, kidney samples were paraffin-embedded and cut into 4-μm sections, followed by deparaffinization and rehydration. Kidney sections were pretreated with proteinase K and then incubated with TUNEL reaction mixture for 1 h at 37 °C. The number of TUNEL-positive cells was counted in five randomly selected non-overlapping fields in each kidney sample under 400× magnification.

### 3.9. Immunohistochemistry (ICH) Staining

Immunohistochemistry was performed on a Dako Autostainer (Dako, Carpinteria, CA, USA). Slides were incubated with 3% hydrogen peroxide and 2.5% normal horse serum (S-2012, Vector Laboratories, Burlingame, CA, USA), followed by incubation with rabbit polyclonal anti-collagen I primary antibody diluted 1:200 in 2.5% normal horse serum for 60 min. Signals were detected with an anti-rabbit IgG Polymer Detection Kit (MP-7401, Vector Laboratories, Burlingame, CA, USA). Labeling was visualized with 3,3′-diaminobenzidine as the chromogen (SK-4105, Vector Laboratories, Burlingame, CA, USA). Slides were counterstained with Harris hematoxylin (EK Industries, Joliet, IL, USA), and whole-slide digital images were collected at 400× magnification with an Aperio Scan Scope slide scanner (Aperio, Vista, CA, USA).

### 3.10. Cell Culture and Treatment

Human proximal tubule (HK-2) cells were purchased from the Cell Bank of the Chinese Academy of Sciences (Shanghai, China) and cultured in DMEM (glucose content, 5.5 mM; Gibco, Shanghai, China), supplemented with 10% fetal bovine serum, and 1% penicillin/streptomycin in a 5% CO_2_ atmosphere at 37 °C. For all experiments, the cells were plated at an appropriate density according to the experimental design and were grown for 24 h to reach 70–80% confluence before experimentation. The groups were as follows: (1) BSA group (control); (2) BSA + NGR1 group (NGR1); (3) AGE-BSA group (AGEs); (4) AGE-BSA + NGR1 (AGEs + NGR1).

### 3.11. Cell Viability Assay

An MTT (3-(4-5-dimethylthiazol-2-yl-2,5-diphenyltetrazolium bromide) assay was employed to determine the cell viability of the HK-2 cells. Cells cultured in 96-well plates (1 × 10^4^ cells/well) were incubated with MTT solution (1 mg/mL final concentration) at 37 °C for 4 h after the various treatments. The formazan crystals were dissolved with dimethyl sulfoxide (DMSO, 150 μL/well), and the absorbance was detected at 570 nm on a microplate reader. Cell viability was expressed as the percentage of MTT reduction compared with the control conditions.

### 3.12. Detection of Mitochondrial Superoxides (ROS)

MitoSOX Red (Molecular Probes, Eugene, OR, USA), a mitochondrial superoxide indicator, was employed to determine mitochondrial superoxide production in HK-2 cells. Briefly, after treatment, the cells were washed once with PBS and incubated with MitoSOX Red (5 μM) in the dark at 37 °C for 10 min. The cells were washed with PBS and then observed under a fluorescence microscope. The fluorescence of MitoSOX Red was measured using a microplate reader (Spectrafluor, TECAN, Sunrise, Austria) at the excitation and emission wavelengths of 510 nm and 580 nm, respectively.

### 3.13. Detection of Mitochondrial Membrane Potential (∆Ψm)

JC-1 (Invitrogen, Waltham, MA, USA) was used to detect the alteration in mitochondrial transmembrane potential (MTP). Briefly, HK-2 cells were incubated with JC-1 (5 μmol/L) at 37 °C for 30 min and were then washed with PBS followed by image acquisition using fluorescence microscopy (DM4000B, Leica, Wetzlar, Germany).

### 3.14. Flow Cytometric Detection of Apoptosis

After the HK-2 cells were treated with NGR1 and AGEs, apoptosis was determined using an Annexin V fluorescein isothiocyanate (FITC)/PI apoptosis kit according to the manufacturer’s instructions (Invitrogen, Waltham, MA, USA). In brief, the HK-2 cells were harvested, washed twice with cold PBS, incubated with the 5 μL of FITC/Annexin V and 1 μL of PI working solution (100 μg/mL) for 30 min in the dark at room temperature, and cellular fluorescence was detected by flow cytometry analysis.

### 3.15. Western Blotting Analysis

Cytoplasmic and nuclear protein samples were separated by protein extraction kits containing 1% phenylmethylsulfonyl fluoride (CoWin Bioscience Co., Ltd., Beijing, China). Western blots were performed using a standard blotting protocol, as described previously [56]. Equal amounts (40 μg) of protein fractions were separated by electrophoresis on 10% SDS-PAGE gels, and transferred into nitrocellulose membranes (Millipore Corporation, Bedford, MA, USA) in Tris–glycine buffer at 300 mA for 1 h. The membranes were blocked with 5% (*w*/*v*) nonfat milk powder in Tris-buffered saline containing 0.1% (*v*/*v*) Tween-20 (Sigma-Aldrich, St. Louis, MO, USA, TBST) and then incubated overnight with appropriate primary antibodies at 4 °C. Afterward, they were washed three times with TBST and incubated with secondary antibodies for 2 h at room temperature. The results were visualized by enhanced chemiluminescence.

### 3.16. Statistical Analysis

Data from at least three independent experiments were expressed as the means ± SD. Statistical comparisons between different groups were measured using one-way ANOVA followed by the Student–Newman–Keuls test. The level of significance was set at *p* < 0.05.

## 4. Conclusions

In summary, we herein demonstrated for the first time that NGR1 promotes Nrf2-mediated HO-1 expression to prevent diabetic nephropathy. Our research indicates that NGR1 may have potential therapeutic properties for the treatment of diabetic nephropathy beyond glucose control.

## Supplementary Materials

The following are available online at http://www.mdpi.com/1420-3049/24/2/247/s1: Figure S1: Impact of NGR1 on diabetes-induced apoptosis in kidney tissue stained by TUNEL.
