# Supplementary material for: Notoginsenoside R1 Protects db/db Mice against Diabetic Nephropathy via Upregulation of Nrf2-Mediated HO-1 Expression
Source: Molecules. 2019 Jan 10;24(2):247. doi: 10.3390/molecules24020247 (PMC6359411; doi:10.3390/molecules24020247)
Supplement: Supplementary file 1 [file molecules-24-00247-s001.pdf]

molecules

Supplementary Material

Notoginsenoside R1 Protects *db/db* Mice against Diabetic Nephropathy via Upregulation of Nrf2-Mediated HO-1 Expression

Bin Zhang [1,2,3,4,†], Xuelian Zhang [1,2,3,4,†], Chenyang Zhang [1,2,3,4], Qiang Shen [1], Guibo Sun [1,2,3,4,*] and Xiaobo Sun [1,2,3,4,*]

1 Institute of Medicinal Plant Development, Peking Union Medical College and Chinese Academy of Medical Sciences, Beijing 100193, China; zhangbin7@126.com (B.Z.); zxl2022@126.com (X.Z.); zcy2022@126.com (C.Z.); qshen666@126.com (Q.S.)

2 Key Laboratory of Bioactive Substances and Resources Utilization of Chinese Herbal Medicine, Ministry of Education, Beijing 100193, China

3 Beijing Key Laboratory of Innovative Drug Discovery of Traditional Chinese Medicine (Natural Medicine) and Translational Medicine, Beijing 100193, China

4 Key Laboratory of efficacy evaluation of Chinese Medicine against Glycolipid Metabolism Disorder Disease, State Administration of Traditional Chinese Medicine, Beijing 100193, China

* Correspondence: sunguibo@126.com (G.S.); sun_xiaobo163@163.com (X.S.); Tel.: +86-015-783-3220 (G.S.); +86-015-783-3013 (X.S.)

† These authors contributed equally to this work.

Received: 2 October 2018; Accepted: 3 January 2019; Published: 10 January 2019

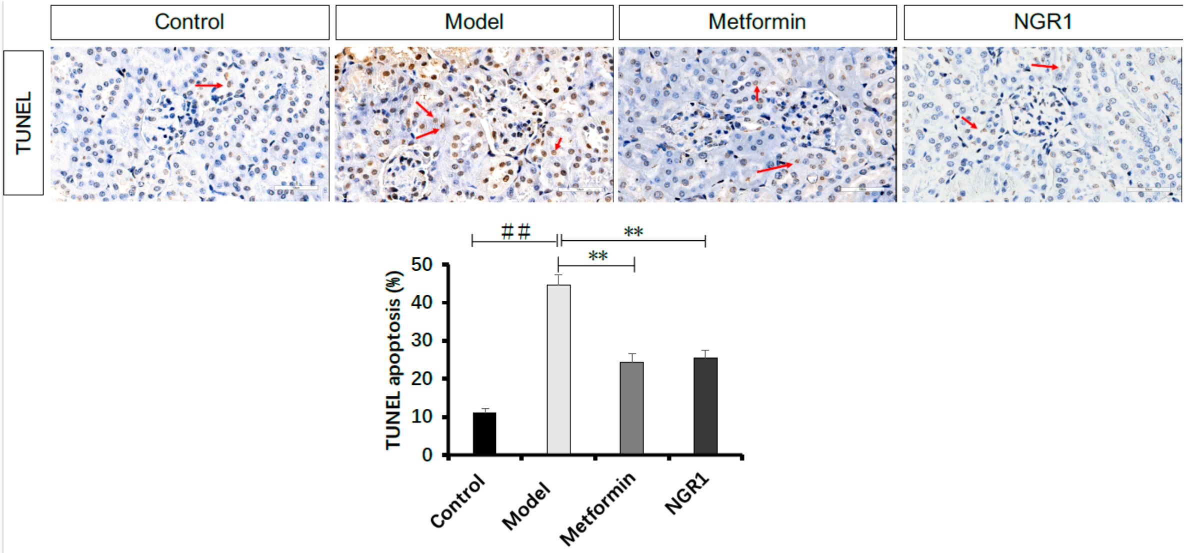

Figure S1. Impact of NGR1 on diabetes-induced apoptosis in kidney tissue stained by TUNEL; (**A**) and the graph showing the percentage of apoptosis within group; (**B**). TUNEL, terminaldeoxynucleotidyl transferase (TdT) dUTP nick-end labeling. ## $p < 0.01$ vs. the control group; ** $p < 0.01$ vs. the model group.

© 2019 by the authors. Submitted for possible open access publication under the terms and conditions of the Creative Commons Attribution (CC BY) license (http://creativecommons.org/licenses/by/4.0/).
